# Acupuncture vs. antispasmodics in the treatment of irritable bowel syndrome: An adjusted indirect treatment comparison meta-analysis

**DOI:** 10.3389/fphys.2022.1001978

**Published:** 2022-10-06

**Authors:** Yun-zhou Shi, Qing-feng Tao, Di Qin, Min Chen, Shu-guang Yu, Hui Zheng

**Affiliations:** ^1^ Department of Acupuncture and Moxibustion, Chengdu University of Traditional Chinese Medicine, Chengdu, China; ^2^ The Affiliated Hospital of Chengdu University of Traditional Chinese Medicine, Chengdu, China

**Keywords:** acupuncture, antispasmodics, irritable bowel syndrome, treatment comparison, meta-analysis

## Abstract

**Background:** Acupuncture has been extensively applied to manage irritable bowel syndrome (IBS) in clinical practice in China. Some randomized controlled trials (RCTs) have demonstrated their efficacy, but it has rarely been compared with first-line antispasmodics to verify their effectiveness. Therefore, we compare acupuncture with antispasmodics in the treatment of IBS by using an adjusted indirect treatment comparison meta-analysis.

**Methods:** Embase, OVID Medline, and the Cochrane Central Register of Controlled Trials databases were searched from inception to 14 March 2022, with no language restrictions. RCTs comparing antispasmodics or acupuncture with placebo or one of the antispasmodics were enrolled. The primary outcome of interest was the improvement of abdominal pain. And the secondary outcomes of interest were the relief of global IBS symptoms and adverse events. The random-effects model was utilized to pool data. The effect size was measured by standardized mean difference (SMD) or relative ratio, and the effectiveness of acupuncture and different antispasmodics were ranked by P-scores.

**Results:** Thirty-five RCTs (n = 5,190) were included. The analysis showed that cimetropium, drotaverine, acupuncture, and pinarverium were superior over placebo in relieving abdominal pain; cimetropium (SMD, −3.00 [95%CI, −4.47 to −1.53], P-score = 0.99) ranked the most effective. In pairwise comparisons, acupuncture had a greater improvement than most antispasmodics except cimetropium and drotaverine in relieving abdominal pain, although the between-group difference was statistically insignificant. In the analysis of continuous outcome in the relief of global IBS symptoms, the result showed that pinaverium was more effective (SMD, 1.72 [95%CI, 0.53 to 2.92], P-score = 0.90) than placebo. Trimebutine and acupuncture had greater improvements than placebo, but no significant difference was shown between groups. In pairwise comparisons, acupuncture was more effective than pinaverium (SMD, −1.11 [95%CI, −1.94 to −0.28]) in relieving global IBS symptoms. In the analysis of adverse events, acupuncture had a lower adverse event rate than most of the other antispasmodics.

**Conclusion:** Cimetropium, drotaverine, and acupuncture were all better than placebo in improving abdominal pain. Acupuncture was preferred over pinaverium in relieving global IBS symptoms, and acupuncture had lower adverse events than most antispasmodics.

## Introduction

Irritable bowel syndrome (IBS) is a commonly functional gastrointestinal disorder characterized by abdominal pain associated with changes in stool form and/or frequency (Drossman and Hasler, 2016). The relative surveys had shown IBS prevalence was 16.8%. Participants suffering from IBS were characterized by a higher prevalence of psychiatric diagnosis and sleep disturbances, higher levels of job strain and isostrain as well as by lower levels of workability compared to non-affected subjects (Buselli et al., 2021). Because the mechanisms of IBS are multifactorial and complicated (Ford et al., 2020), IBS is challenging to manage and has a significant impact on social functioning and quality of life (Frändemark et al., 2018). The previous evidence showed that after 1 and 7 years, over 50% of patients with IBS had the same symptoms and a further one-quarter of patients reported consistent mild IBS symptoms (Agréus et al., 2001). At the same time, based on a burden of disease study, it was reported that the costs directly attributable to IBS in the USA were estimated at US$1 billion and indirect costs were as high as $50 million (Everhart and Ruhl, 2009). Currently, the Rome IV criteria recommend that the choice of treatment should focus on the major symptomatology. According to predominant bowel habits, the pharmacological therapies include soluble fiber (Galica et al., 2022), antispasmodic drugs (Annaházi et al., 2014), central neuromodulators (Fadgyas Stanculete et al., 2021), intestinal secretagogues (Brenner et al., 2018), drugs acting on opioids, or 5-HT receptors (Jones et al., 2021), or minimally absorbed antibiotics (Li et al., 2016). Because antispasmodics were available for all subtypes of IBS, and accumulating evidence showed that antispasmodics can effectively and safely regulate gastrointestinal motility disturbances, improve bowel habits, and relieve abdominal pain/discomfort (Ruepert et al., 2011; Martínez-Vázquez et al., 2012). The international clinical practice guidelines recommended that antispasmodics were considered as the first-line therapy for IBS (Moayyedi et al., 2019).

In China, acupuncture has been extensively applied to manage gastrointestinal diseases in clinical practice. The relative evidence reported that acupuncture can regulate the bowel characteristics and frequency of stool for IBS (Manheimer et al., 2012a). Nevertheless, throughout the past 20 years, the studies of acupuncture concentrated primarily on the specific effect of acupuncture vs. sham acupuncture. Although previous trials investigated the impact of acupuncture on IBS, the majority of trials compared acupuncture with a placebo, and only a minority of them directly compared acupuncture with conventional treatments such as antispasmodics. Three randomized controlled trials (RCTs) which compared acupuncture with pinaverium showed that acupuncture seemed to be more effective in alleviating abdominal pain, improving stool frequency, and reducing the recurrence rate for the management of IBS (Li et al., 2012; Pei et al., 2020). However, the relative study between acupuncture and other antispasmodics such as cimetropium or drotaverine was scarce.

Regarding that antispasmodics have been conventionally prescribed as the first-line drug for IBS (Quigley et al., 2016), it is essential to compare acupuncture and antispasmodics to verify the effectiveness of acupuncture. Therefore, we conducted an adjusted indirect meta-analysis to compare acupuncture with antispasmodics in order to confirm whether acupuncture was equally effective to antispasmodics in the management of IBS.

## Methods

### Study source

We searched the following three electronic databases from inception to 14 March 2022: Embase, OVID Medline, and the Cochrane Central Register of Controlled Trials (CENTRAL). RCTs comparing antispasmodics or acupuncture with placebo or one of the antispasmodics were included. Furthermore, Clinical registries (Clinicaltrials.gov) and published systematic reviews were also searched for any missed RCTs. Besides, we did not limit the language type in our search. A list of search strategies can be found in Supplementary Table S1.

### Study selection

Two investigators independently reviewed the abstract and title, and read the full text in detail to identify included articles. The articles met the following criteria: 1) Diagnostic criteria were limited to Rome I, II, III, and IV criteria; 2) RCTs comparing antispasmodics or acupuncture with placebo or one of the antispasmodics were included; 3) Adult patients with IBS were included; 4) The treatment duration and dose range were limitless, but the interventions were provided for at least a week; 5) At least one of the targeted outcome measurement listed below was required to be obtainable: global IBS symptoms, abdominal pain, or adverse events; 6) RCTs with both inflammatory bowel diseases and IBS were included if IBS date were independently showed. We harmonized any disagreements by consensus and finally judged by a third investigator.

### Outcome assessments

The primary outcome of interest was the improvement of abdominal pain. And the secondary outcome of interest was the relief of global IBS symptoms. We also assessed the number of treatment-related adverse events for the safety outcome.

### Data collection

According to a standardized form, one investigator extracted the descriptive data, which was then verified by another investigator. We abstracted the following data from the included study: author, published year, study design, the proportion of female participants, mean age, diagnostic criteria, IBS subtype, details of interventions and controls, and outcome data. The number of participants and corresponding events was presented with dichotomous data, while continuous data was presented as mean and standard deviation.

### Risk of bias assessment

The risk of bias of each RCT was evaluated by the second edition of the Cochrane risk of bias (RoB 2.0) (Sterne et al., 2019). Each study was assessed in five parts with certain questions in RoB 2.0, finally, the overall risk of bias for the study was judged to be low, some concerns or high. Besides, we also utilized the GRADE system to evaluate the confidence level of evidence in this study. And according to the quality assessment of study design, risk of bias, indirectness, inconsistency, imprecision, and other consideration, the evidence was classified into four levels: high quality, moderate quality, low quality, or very low quality.

### Data synthesis

We used the frequentist method to conduct this indirect treatment comparison meta-analysis (Rücker, 2012). We plotted net graphs to identify direct and indirect comparisons between acupuncture and different antispasmodics. A random-effects model was applied to compare the included treatments in this meta-analysis. Forest plots were drawn according to the different outcomes, showing acupuncture or antispasmodics vs. placebo, and displaying the effect sizes and their associated 95% confidence intervals (95%CIs). We calculated the relative ratio (RR) for categorical outcomes including the relief responder rate and adverse event rate. If the relative study with zero events in an arm, it was excluded from the analysis (Mills et al., 2013). For continuous outcomes such as the change in abdominal pain and the improvement of global IBS symptoms, we used the standardized mean difference (SMD). If continuous outcomes were evaluated at different time points, they were combined by using a multivariate analysis (Riley et al., 2017). Besides, the effectiveness of acupuncture and different antispasmodics were ranked by the P-scores method (Rücker and Schwarzer, 2015). P-scores were based solely on the point estimates and standard errors of the frequentist network meta-analysis estimates under normality assumption. They measure the mean extent of certainty that a treatment was better than the competing treatments. The consistency of the network was checked by comparing network estimates, indirect, and direct. And we examined the implications of inconsistency through the use of the z-test. In addition, the transitivity assumption in this study was evaluated through the global heterogeneity, which was estimated by calculating the tau-squared statistics and global I^2^. The global heterogeneity was categorized into three levels of small, large, and very large according to the cutoff point of I^2^<50%, 50% ≤ I^2^<75%, and I^2^ ≥ 75%, respectively. When large heterogeneity emerged, we conducted a design-by-treatment analysis by decomposing Cochran’s Q to identify the reason for heterogeneity (Krahn et al., 2013).

## Results

### Trial characteristics

**FIGURE 1:**
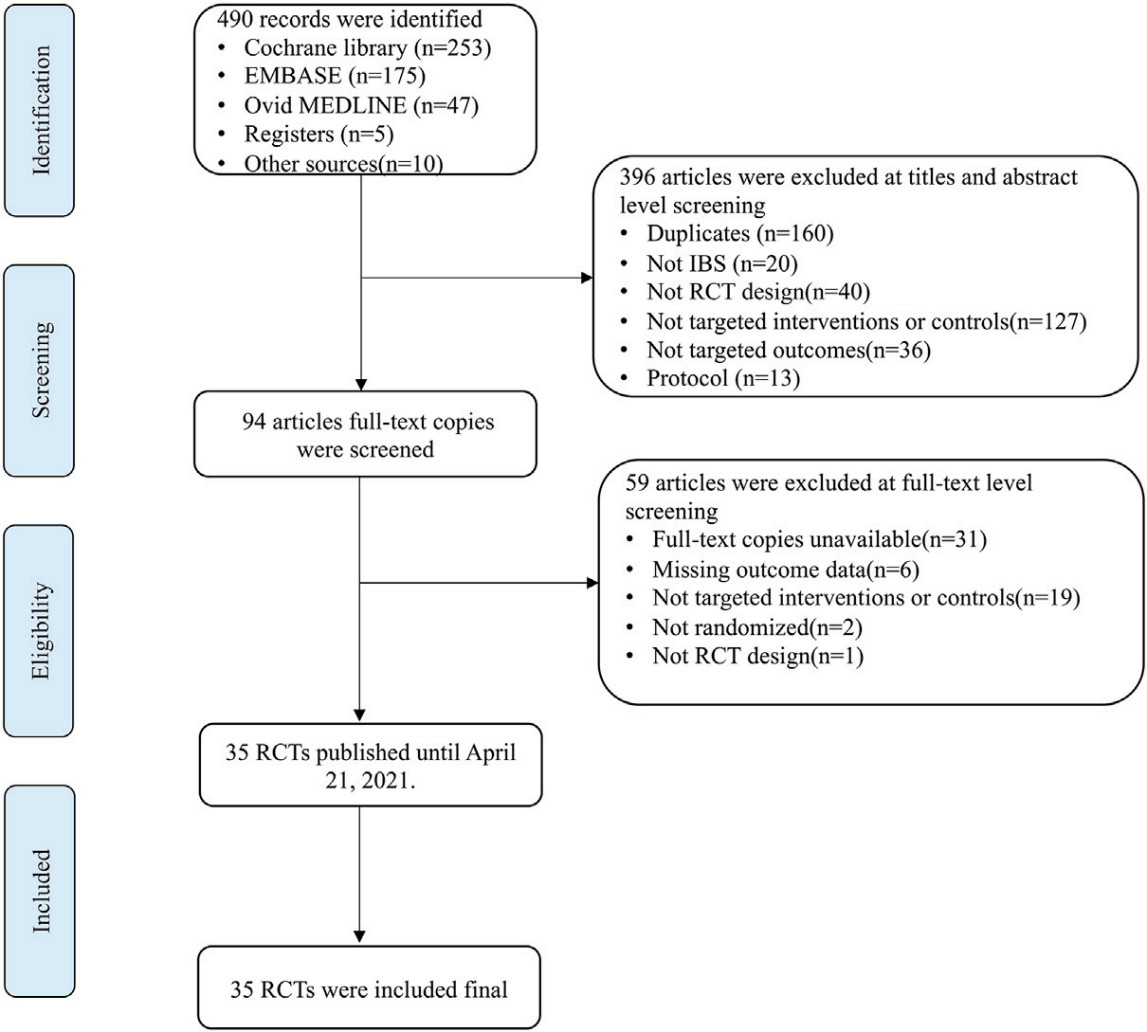
Study flowchart.

A total of 490 articles were detected in the initial search. 160 duplicate articles were excluded by using Zotero and manual searches. After screening the titles and abstracts, 236 articles were excluded. Then, full-text copies were screened based on the inclusion and exclusion criteria, and 59 articles were further excluded. Ultimately, 35 articles were included in this study. The flow of studies through the selection process is presented in Figure 1.

In the 35 articles, 27 assessed the effects of antispasmodics (3,999 participants), and 8 RCTs assessed acupuncture (1,191 participants). The median age of included participants was 35.6 years (range, 26–51.6 years), and the percentage of females was 59%. The detailed characteristics of interventions, controls, and outcomes of the included 35 articles are shown in Table 1.

**TABLE 1:** Trial characteristics.

Study ID	Design	Sample size	Mean age (years)	Female (%)	Diagnostic criteria	IBS subtypes	Interventions	Study period (wks)	Outcomes
Anastasi, 2009	Single-center	29	40.4	66	Rome criteria	Not mentioned	Acupuncture 2 times/week	4	IBS-GIS; Abdominal pain/discomfort
Battaglia, 1998	Multicenter	325	47.7	69	Rome I	Not mentioned	Otilonium 40 mg tds	15	Abdominal pain; Global assessment
Centonze, 1988	Single-center	48	NA	50	NA	Not mentioned	Cimetropium 50 mg tds	24	Abdominal pain; Global assessment
Chakraborty, 2019	Single-center	40	35.6	75	Rome IV	IBS-D	Mebeverine 200 mg bid	8	Abdominal pain; IBS-QOL
Chmielewska-Wilkoń, 2014	Multicenter	93	44.8	64	Rome II	Not mentioned	Otilonium 20 mg tid	4	Abdominal discomfort; Intestinal habits and global discomfort; Adverse effect
Clavé, 2011	Multicenter	356	46.6	71	Rome II	Not mentioned	Otilonium 40 mg tid	15	Abdominal pain; IBS symptom scale
Connell, 1965	NA	40	40	63	NA	all subtype	Mebeverine 400 mg	12	Adverse effect; Global assessment
Dobrilla, 1990	Single-center	70	45	67	Not defined	all subtype	Cimetropium 50 mg tid	12	Global symptoms
Everitt, 2013	Multicenter	135	44	80	Rome III	Not mentioned	Mebeverine 135 mg tid	6	IBS symptom scale; IBS-QOL
Fielding, 1980	NA	60	26	75	Not defined	Not mentioned	Trimebutine 200 mg tds	24	Abdominal pain; Global assessment
Forbes, 2005	Single-center	59	43.7	66	Rome I; RomeⅡ; Manning criteria	Not mentioned	Acupuncture 10 times	13	Global symptoms; HAD; EuroQol instruments
Ghidini, 1986	Single-center	60	NA	60	NA	Not mentioned	Rociverine/Trimebutine tid	8	Abdominal pain
Gilvarry, 1989	NA	24	32	79	Not defined	Not mentioned	Pirenzepine 100 mg	4	Abdominal pain; Global assessment
Glende, 2002	Multicenter	317	44	69	Rome I	Not mentioned	Otilonium 40 mg tid	15	Abdominal pain
Kruis, 1986	Single-center	80	41	61	NA	all substyle	Mebeverine 100 mg 4 dd	16	Abdominal pain; Global assessment
Lembo, 2009	Single-center	262	38.5	76	Rome II	Not mentioned	Acupuncture 2 times/week	3	IBS-GIS; IBS-AR; IBS-SSS; IBS-QOL
Li, 2013	Single-center	70	38.5	47	Rome III	IBS-D	Acupuncture 3–4 times/week	4	Global assessment; IBS-QOL
Li, 2017	Multicenter	81	47	61.7	Rome III	IBS-D	Acupuncture 3 times/week	6	IBS-SSS; PSQI; Global assessment; Adverse effect
Lowe, 2017	Single-center	79	43	79	Rome I	Not mentioned	Acupuncture 1 times/bi-week	4	Global assessment; SF-36; IBS-36 QOL tools; McGill pain score; PSQI
Lüttecke, 1980	Single-center	40	45.3	53	NA	Not mentioned	Trimebutine 200 mg tid	1	Global symptoms
Mak, 2019	Single-center	80	51.6	53	Rome III	IBS-D	Electroacupuncutre 1 times/week	10	Bowel symptoms; Somatic symptoms; Health-related quality of life
Mitchell, 2002	Multicenter	107	53	80	Rome I	Not mentioned	Alverine 360 mg	12	Abdominal pain; Global assessment
Moshal, 1979	Single-center	20	27	35	Not defined	Not mentioned	Trimebutine 200 mg tds	4	Abdominal pain
Page, 1981	Multicenter	97	36.7	83	NA	Not mentioned	Dicyclomine 40 mg qid	2	Abdominal pain; Global assessment
Passaretti, 1989	Single-center	40	39	60	NA	Not mentioned	Cimetropium 50 mg tds	4	Abdominal pain; Global assessment
Pei, 2020	Multicenter	531	46.4	47.5	Rome III	IBS-C, IBS-D	Acupuncture 3 times/week	6	IBS-SSS; IBS-QOL; Adverse effect
Piai, 1979	Single-center	18	NA	56	Not defined	Not mentioned	Prifinium 30 mg tds	6	Global assessment
Rai, 2014	Multicenter	180	46.5	13	Rome II	Not mentioned	Drotaverine 80 mg tid	4	Abdominal pain; Bristol stool form scale
Schäfer, 1990	Multicenter	360	NA	NA	NA	Not mentioned	Butylscopamine 30 mg	4	Abdominal pain; Global assessment
Wittmann, 2010	Multicenter	412	46.2	71	Rome III	Not mentioned	ACS tid	4	Abdominal pain; IBS symptom scale
Xue, 2017	Single-center	144	43.2	65	Rome II	all subtype	Drotaverine 80 mg tid	4	Abdominal pain; Stool frequency; Bristol scale; SF-36; Adverse effect
Yuan, 2005	Multicenter	160	NA	NA	Rome II	Not mentioned	Trimebutine 200 mg tid	4	Global assessment
Zheng, 2015	Multicenter	427	36.7	55	Rome III	Not mentioned	Pinaverium 50 mg tid	4	Abdominal pain; Bristol stool form scale
Zheng, 2021	Multicenter	264	39.9	60	Rome IV	IBS-D	Pinaverium 50 mg tid	4	Pain intensity; Bristol stool form scale
Zhong, 2009	Single-center	82	36.6	52	Rome III	IBS-D	Alverine 60 mg bid	8	Abdominal pain

Notes: ACS, alverine citrate 60 mg + simeticone 300 mg.

**FIGURE 2:**
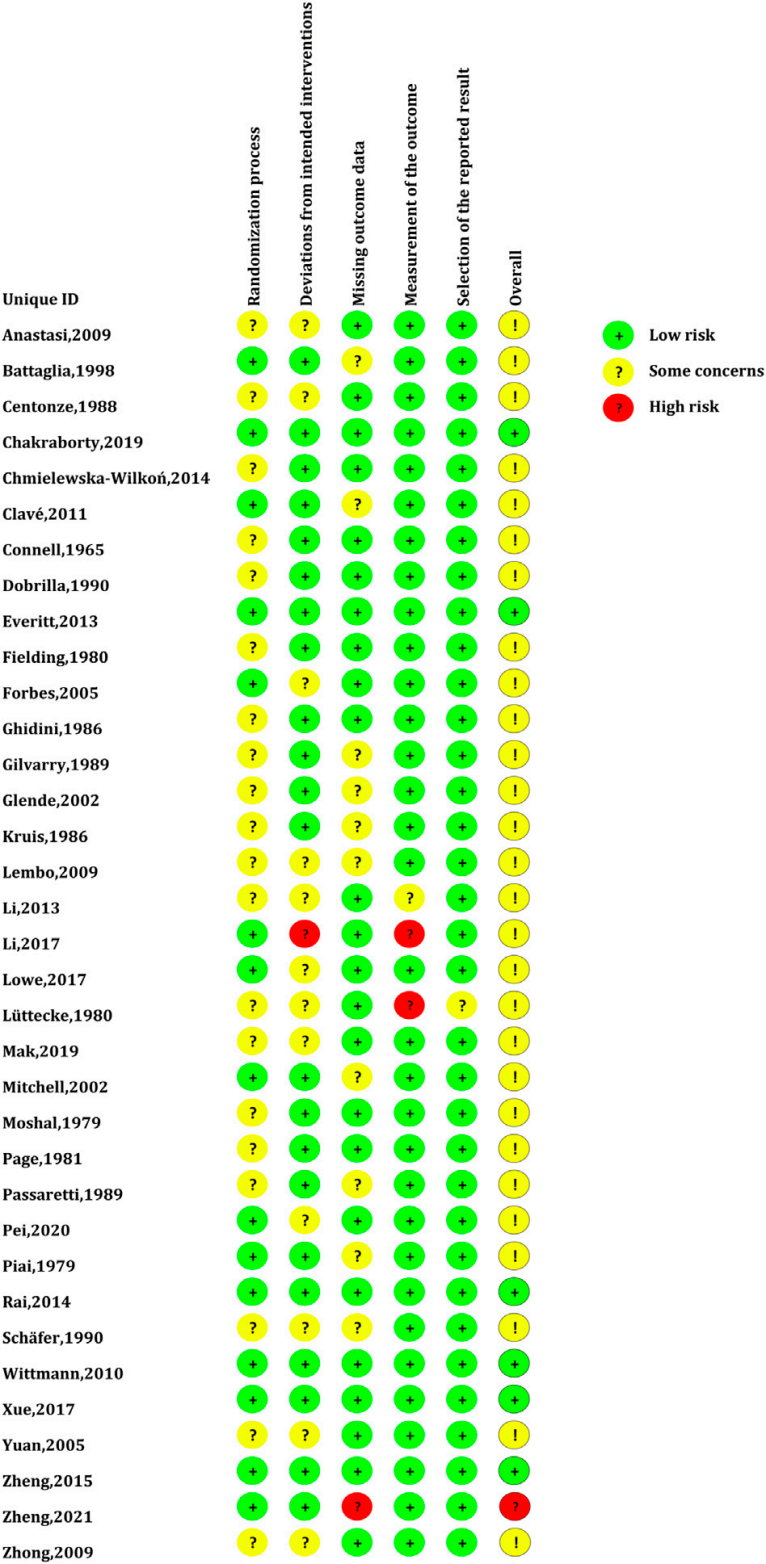
Risk of bias assessment for individual RCT.

The global risk-of-bias assessment demonstrated that there were 6 RCTs (17.14%) with a low risk of bias, 28 (80%) RCTs with a moderate risk of bias, and 1 RCT (2.86%) with a high risk of bias. The risk of bias assessment for individual RCT is presented in Figure 2.

GRADE assessment demonstrated that the comparative quality of acupuncture vs. antispasmodics was very low for the improvement of abdominal pain and global IBS symptoms, and the adverse events were of low quality. The summary of findings is shown in Supplementary Table S2.

### The improvement of abdominal pain

**FIGURE 3:**
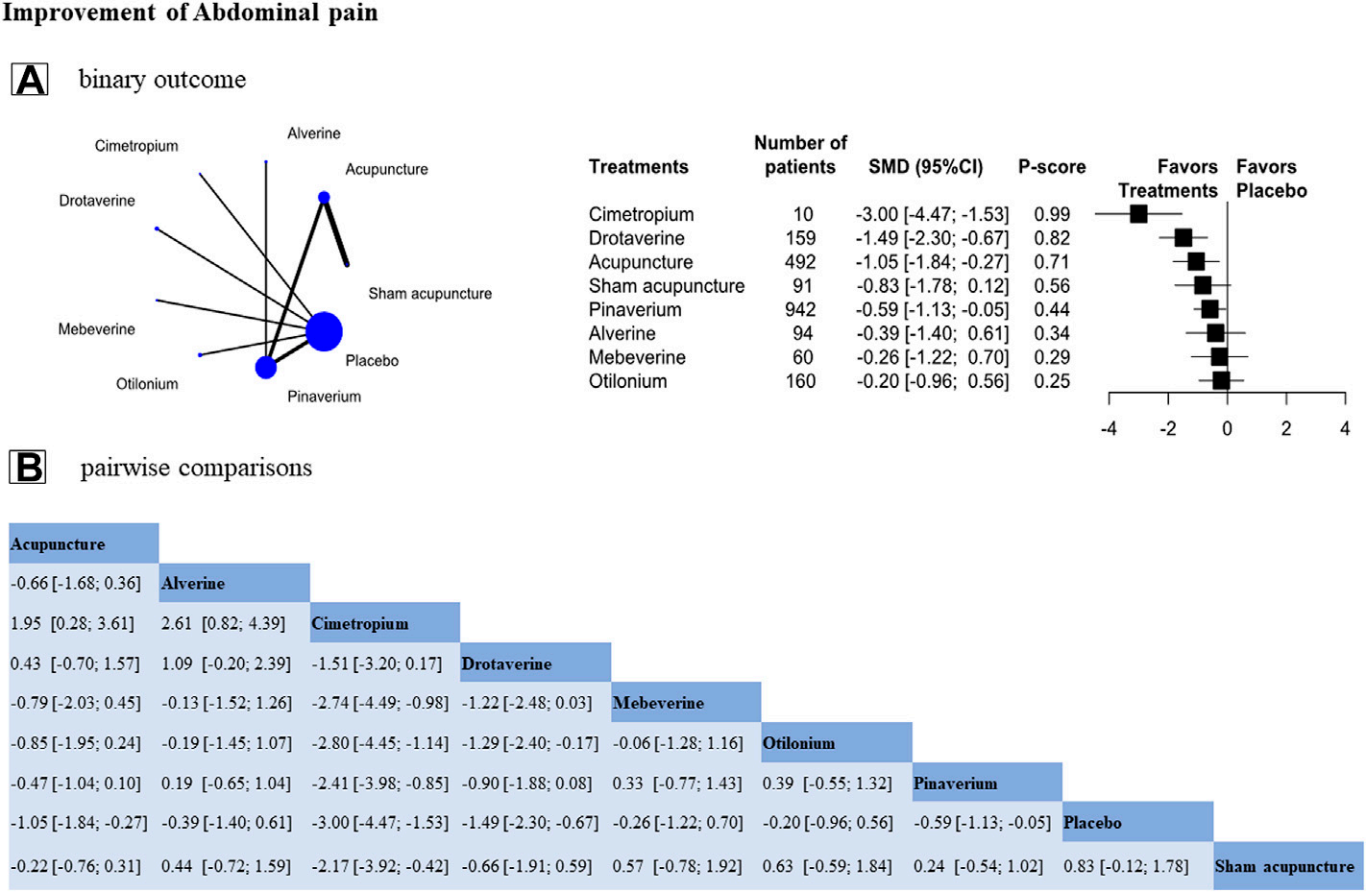
Individual-level comparison of the improvement of abdominal pain. Footnote: Individual-level analysis results **(A)** and the pairwise comparisons results **(B)** are shown in this figure **(A)**: The geometry of the networks is shown on the left. The size of the blue nodes corresponds to the number of participants assigned to treatments. The right shows the forest plots using placebo as a reference. Direct comparison was linked by a line between two treatments; the thickness of the lines corresponds to the number of trials that studied the treatment. P-scores are used to rank the effectiveness of each treatment. Treatments with the highest *p* values are the most effective. Standardized mean difference (SMD) > 0 means this treatment superiority over placebo **(B)**: A comparison estimate and its 95% confidence intervals (95% CI) are in the cell between column-defining treatment and row-defining treatment. The upper triangle shows pairwise comparisons of column-defining treatment vs. row-defining treatment. The lower triangle shows pairwise comparisons of row-defining treatment vs. column-defining treatment. For abdominal pain in the upper triangle, SMD>0 favors row-antispasmodics, SMD<0 favors column-acupuncture.

This analysis contained 9 RCTs (n = 2086), and assessed eight treatments in total; 4 RCTs evaluated the effectiveness of antispasmodics, and 5 RCTs assessed acupuncture. The result showed that cimetropium (SMD, −3.00 [95%CI, −4.47 to −1.53], P-score = 0.99, global I^2^ = 82.2%, Figure 3A) was ranked as the most effective treatment. Drotaverine, acupuncture, and pinarverium were superior to placebo (Figure 3A). In pairwise comparisons, cimetropium was better than most other treatments in relieving abdominal pain, except drotaverine. Compared with antispasmodics, acupuncture had a greater improvement than most antispasmodics except cimetropium and drotaverine in relieving abdominal pain, but the between-group difference was not significant (Figure 3B).

### The relief of global IBS symptoms

**FIGURE 4:**
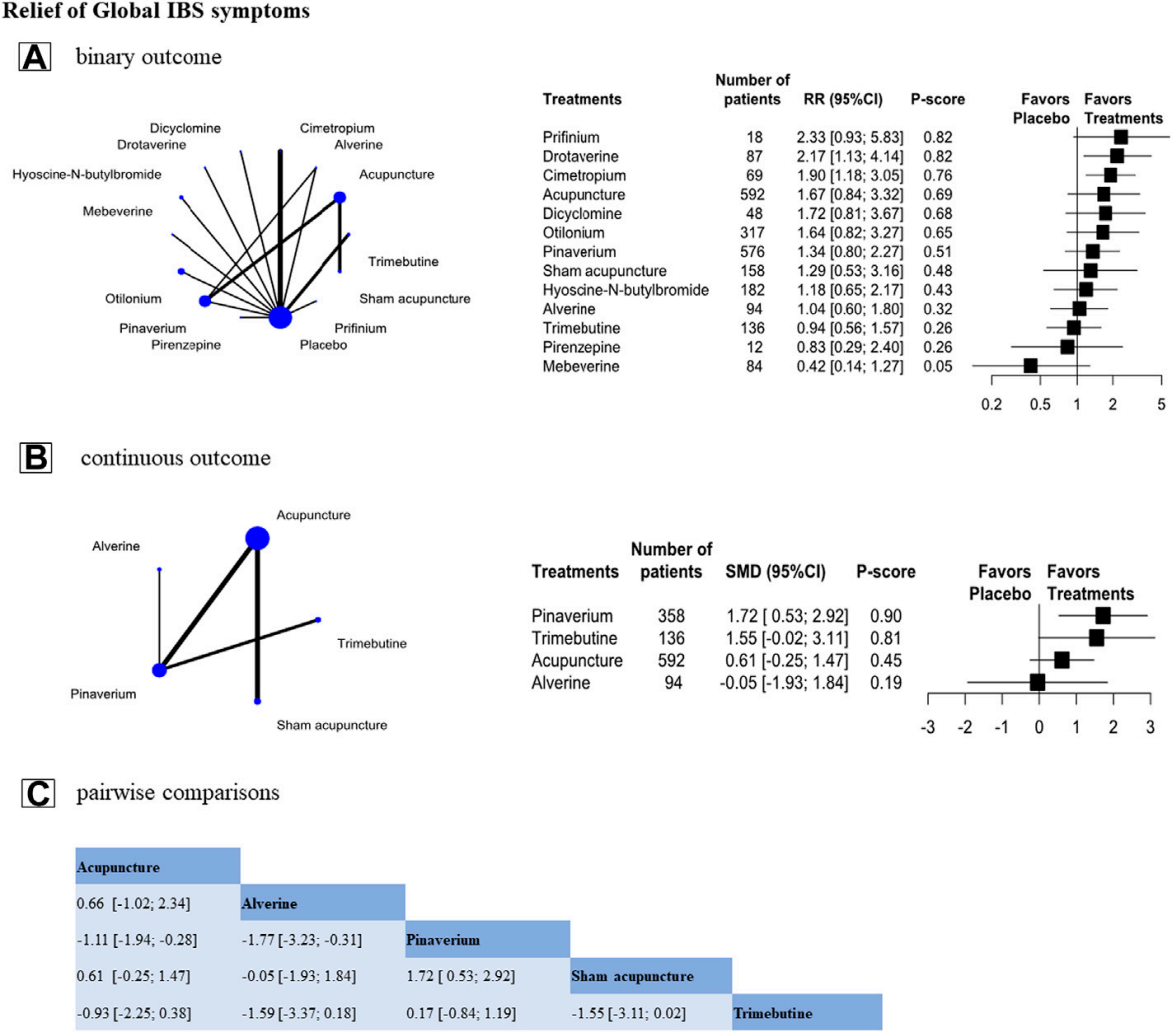
Individual-level comparison of the relief of Global IBS symptoms. Footnote: Individual-level binary and continuous outcomes analysis results **(A,B)** and the pairwise comparisons of continuous outcome results **(C)** are shown in this figure **(A,B)**: The geometry of the networks is shown on the left. The size of the blue nodes corresponds to the number of participants assigned to treatments. The right shows the forest plots using placebo as a reference. Direct comparison was linked by a line between two treatments; the thickness of the lines corresponds to the number of trials that studied the treatment. P-scores are used to rank the effectiveness of each treatment. Treatments with the highest *p* values are the most effective. **(A)**: Standardized mean difference (SMD) > 0 means this treatment superiority over placebo. **(B)**: Relative ratio (RR) > 1 means this treatment superiority over placebo **(C)**: A comparison estimate and its 95% confidence intervals (95% CI) are in the cell between column-defining treatment and row-defining treatment. The upper triangle shows pairwise comparisons of column-defining treatment vs. row-defining treatment. The lower triangle of shows pairwise comparisons of row-defining treatment vs. column-defining treatment. For the global IBS symptoms in the upper triangle, SMD>0 favors row-antispasmodics, SMD<0 favors column-acupuncture.

We analyzed 16 RCTs (n = 2,307) on binary outcomes in relieving the symptoms of global IBS and assessed 13 treatments in total (Figure 4A). The result showed that drotaverine (RR, 2.17 [95%CI, 1.13 to 4.14], P-score = 0.82, global I^2^ = 47.4%, Figure 4A) was ranked as the most effective treatment. Drotaverine and cimetropium were better than placebo in relief of global IBS symptoms, and acupuncture also had a greater relief than placebo, but no significant between-group difference was noted (RR, 1.67 [95%CI, 0.84 to 3.32], P-score = 0.69, Figure 4A).

In the analysis of continuous outcome in relieving the symptoms of global IBS, we included eight RCTs (n = 1,072) and assessed 4 treatments (Figure 4B). The result showed that pinaverium was more effective (SMD, 1.72 [95%CI, 0.53 to 2.92], P-score = 0.90, global I^2^ = 89.7%, Figure 4B) than placebo. Trimebutine, acupuncture, and alverine were not superior to placebo (Figure 4B). However, in pairwise comparisons, acupuncture was preferred over pinaverium in relieving global IBS symptoms (SMD, −1.11 [95%CI, −1.94 to −0.28]). But compared to other antispasmodics, acupuncture had a non-significantly in relieving the symptom of global IBS (Figure 4C).

### Adverse events

**FIGURE 5:**
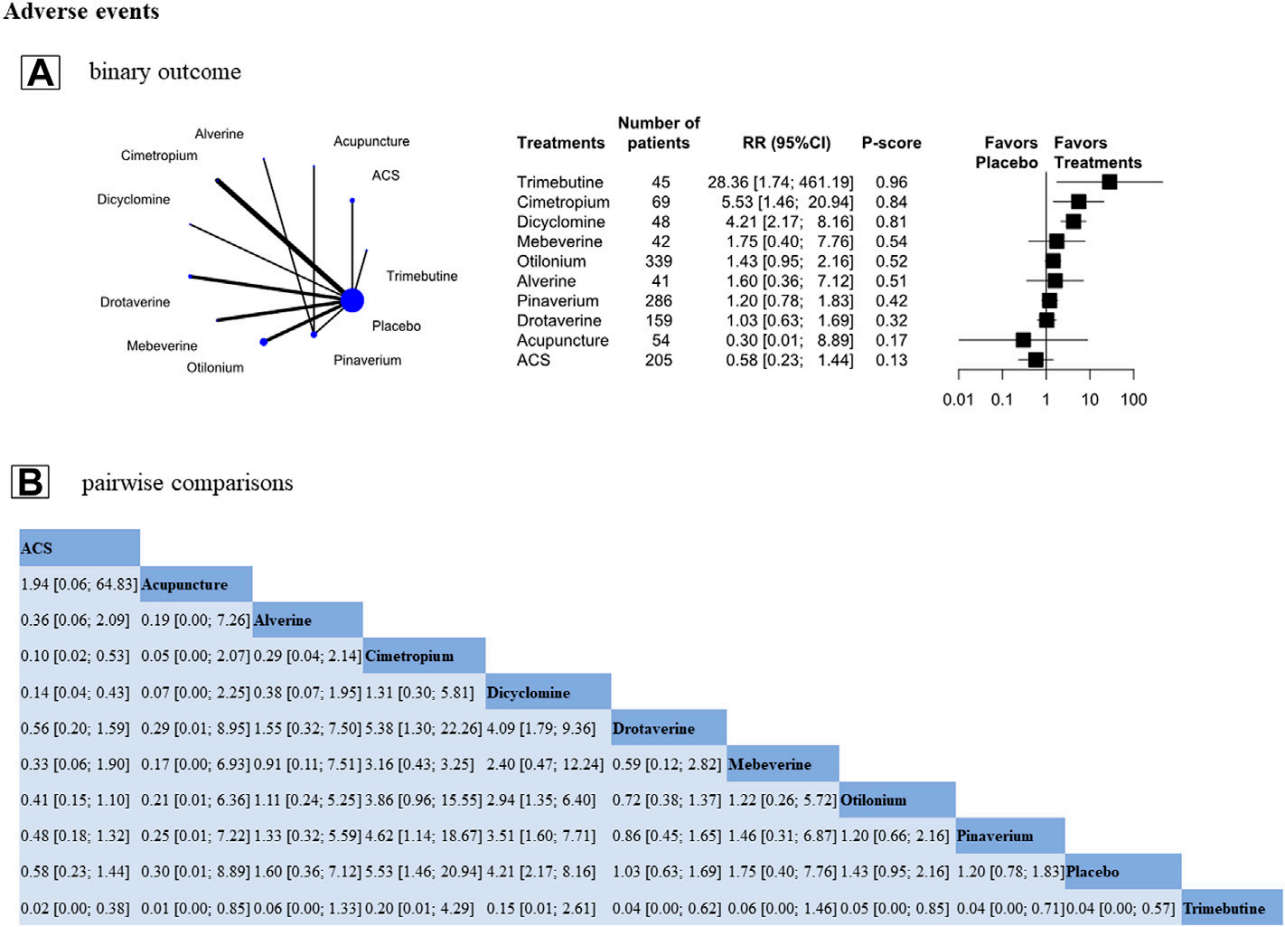
Treatment-related adverse events. Footnote: Individual-level analysis results **(A)** and the pairwise comparisons results **(B)** are shown in this figure **(A)**: The geometry of the networks is shown on the left. The size of the blue nodes corresponds to the number of participants assigned to treatments. The right shows the forest plots using placebo as a reference. Direct comparison was linked by a line between two treatments; the thickness of the lines corresponds to the number of trials that studied the treatment. P-scores are used to rank the adverse events rate of each treatment. Treatments with the highest *p* values are the most adverse events. **(A)**: Relative ratio (RR) > 1 means this treatment high over placebo **(B)**: A comparison estimate and its 95% confidence intervals (95% CI) are in the cell between column-defining treatment and row-defining treatment. The upper triangle shows pairwise comparisons of column-defining treatment vs. row-defining treatment. The lower triangle of shows pairwise comparisons of row-defining treatment vs. column-defining treatment. For the adverse events in the upper triangle, RR > 1 favors row-antispasmodics have a higher adverse events, RR < 1 favors column-acupuncture has a lower adverse events.

The analysis contained 17 RCTs (n = 2,412); 15 RCTs evaluated the effects of antispasmodics, and 2 RCTs assessed acupuncture. The individual-level analysis-assessing 10 treatments-demonstrated that acupuncture had similar adverse event rate to placebo (RR, 0.30 [95%CI, 0.01 to 8.89], P-score = 0.17, global I^2^ = 0%, Figure 5A), and trimebutine (RR, 28.36 [95%CI, 1.74 to 461.19], P-score = 0.96), cimetropium (RR, 5.53 [95%CI, 1.46 to 20.94], P-score = 0.84), dicyclomine (RR, 4.21 [95%CI, 2.17 to 8.16], P-score = 0.81) had higher adverse event rate than placebo (Figure 5A). In pairwise comparisons, acupuncture had a significantly lower adverse events rate than most other treatments (Figure 5B).

In the antispasmodics, the main common adverse events were dry mouth, heartburn sensation, sleepiness, nausea, headache, flatulence, dizziness, or weakness. And the adverse events of acupuncture were slight hematoma around the site of needling and stabbing pain. These conditions were mild, and no medical care was necessary.

## Discussion

As far as we know, this study first used an adjusted indirect treatment comparison method to respond to the clinical issue: Is acupuncture as equivalent as antispasmodics in terms of improving abdominal pain and global IBS symptoms? In this meta-analysis, we found that acupuncture and some antispasmodics (e.g., cimetropium, drotaverine) were better than placebo in improving abdominal pain. In pairwise comparisons, acupuncture was preferred over pinaverium in relieving the symptom of global IBS, and acupuncture had a significantly lower adverse events rate than most other antispasmodics.

The evidence from previous studies about acupuncture for IBS is relatively lacking and contradictory. On one hand, a Cochrane systematic review including17 RCTs reported that the effective rate of acupuncture for IBS was better than pharmacological interventions (Manheimer et al., 2012a), but the pharmacological interventions were sundry and included Chinese herbal formula, probiotics, pinaverium bromide, and sulfasalazine, *etc.* Another systematic review similarly confirmed that at 6-month follow-up, acupuncture was more beneficial for overall symptoms of IBS compared to standard medical treatment (Amsallem et al., 2021). The recent research was validated again and reported that acupuncture may be a more effective treatment than PEG 4000/pinaverium bromide in terms of improving the symptoms of IBS (Pei et al., 2020). Our study partially confirmed this result and showed acupuncture had more beneficial effects than pinaverium in relieving the symptom of global IBS, but compared to other antispasmodics, acupuncture had non-significantly benefits. We considered that this difference may be related to the fact that we did not include other pharmacological interventions except for antispasmodics. On the other hand, another previous study indicated that acupuncture achieved favorable therapeutic benefits, but no statistically significant difference was noted between acupuncture and sham acupuncture (Manheimer et al., 2012b; Lowe et al., 2017). In this study, we also found that acupuncture can improve IBS global symptoms compared with sham acupuncture, but the between-group difference was not significant (SMD, −0.22 [95%CI, −0.76 to 0.31]). Besides, the previous relative study showed that non-specific effects may lead to statistically significant results in the treatment of IBS (Kaptchuk et al., 2008).

The above result indicated that sham acupuncture maybe not be an inert control for IBS. Therefore, the comparison between acupuncture and positive drugs seems to be necessary and able to clearly define the effectiveness of acupuncture for IBS in the future. However, it is worth noting that the GRADE assessment demonstrated that the quality of acupuncture vs. antispasmodics was low for the improvement of abdominal pain and global IBS symptoms. But it does not mean that there is a problem with this research itself, and it is mainly caused by the risk of bias in the included literature, the diversity of antispasmodics, and the nature of indirect comparisons.

As we all know, the mechanisms of IBS are complicated. Relative studies reported that the possible mechanisms included the brain-gut axis, gastrointestinal motility, the immune system, visceral hypersensitivity, and neurotransmitters (Yaklai et al., 2021; Qi et al., 2022). In this study, the result showed that cimetropium, drotaverine, acupuncture, and pinaverium were effective in improving global IBS symptoms. At present, antispasmodics were medications with anticholinergic or calcium channel blocking effects (Annaházi et al., 2014; Chey et al., 2015), which can inhibit intestinal wall contraction and regulate intestinal transport time by improving visceral hypersensitivity and intestinal motility (Martínez-Vázquez et al., 2012; Camilleri, 2018). Some evidence showed that drotaverine had antispasmodic effects on intestinal smooth muscle by inhibiting calcium calmodulin complex and phosphodiesterase enzyme system (Rai and Nijhawan, 2021); Cimetropium was mediated by antagonizing acetylcholine in intestinal smooth muscle muscarinic receptors to achieve antispasmodic effect; Pinaverium was an L-type calcium-channel blocker, which can inhibit calcium influx and prevent colonic smooth muscle cell contractions (Annaházi et al., 2014). However, due to the characteristics of multi-link and multi-target of acupuncture, there is still a lack of in-depth understanding of the mechanisms associated with acupuncture in the treatment of IBS. Relative studies had confirmed that acupuncture can improve visceral hypersensitivity and intestinal motility by regulating the Epac1-Piezo 2 axis and reducing 5-HT and 5-HTR expressions (Zhao et al., 2016; Guo et al., 2022) and regulate the brain-gut axis and nervous system by affecting neurotransmitters including 5-HT, substance P, calcitonin gene-related peptide, nitric oxide, and norepinephrine. In addition, IL-18, IL-23, TNF-α, mast cells, and other immune cells and inflammatory factors may be also involved in the regulation of IBS by acupuncture (Wu et al., 2008; Ma et al., 2014).

Compared to the mechanisms of antispasmodics and acupuncture in the treatment of IBS, we found that both of them can improve intestinal hypersensitivity and intestinal motility. But the mechanisms of acupuncture are relatively complicated and diverse. Several challenges should be resolved before acupuncture was implemented into regular clinical practice. Firstly, A further revelation about these mechanisms of action about acupuncture is of importance to the clinical practice. Secondly, compared to the other pharmacological treatments, acupuncture is a complicated intervention and its therapeutic effects are affected by a series of factors such as needling sensation, acupuncture manipulation, acupoint specificity, psychological factors, and needle duration (Shi et al., 2012). Currently, the parametrization of acupuncture and acupoint selection for the treatment of IBS vary in diverse literature, and until now, there are no relevant studies to explore an optimal, and standardized acupuncture treatment strategy for IBS. Therefore, it is extremely important to use all the above influencing factors for clinical practice to evaluate the true effectiveness of acupuncture for IBS in future studies.

There are some limitations in our study. Firstly, we compared acupuncture with the majority of antispasmodics on the basis of indirect estimates. While the indirect estimates borrowed their power from a variety of sources including direct comparisons of antispasmodics vs. acupuncture, antispasmodics vs. placebo, and acupuncture vs. placebo. It may lead to inaccurately represent the difference between acupuncture and antispasmodics. However, the comparison between pinaverium and acupuncture is consistent for the direct and indirect estimates, which indicated to some extent the credibility of the indirect evidence. Secondly, the analysis of continuous outcomes in the relief of global IBS symptoms and abdominal pain showed slightly greater heterogeneity, the variety of antispasmodics such as different types, dosages, usages, and duration of treatment and different acupuncture prescriptions maybe contribute to the statistical heterogeneity. Thirdly, in order to minimize the selectivity bias of the literature, our inclusion definitions ranged from Rome I to Rome IV. The inclusion criteria are relatively broad, which may lead to cloud the interpretation of data. Fourth, most studies lacked follow-up information for antispasmodics, and we did not evaluate the prolonged effect of acupuncture vs. antispasmodics in the treatment of IBS. But relative studies indicated that acupuncture can continue to improve overall symptoms of IBS lasting for 1–3 months after one-course treatment (Lowe et al., 2017; Mak et al., 2019; Pei et al., 2020). These findings suggested that acupuncture might have a long-term effect and be beneficial in maintaining a sustainable alleviation of IBS symptoms.

## Conclusion

Cimetropium, drotaverine, and acupuncture were all better than placebo in improving abdominal pain. Acupuncture was preferred over pinaverium in relieving global IBS symptoms, and acupuncture had lower adverse events than most antispasmodics.

## Data Availability

The original contributions presented in the study are included in the article/Supplementary Material, further inquiries can be directed to the corresponding author.
