# Supplementary material for: Acupuncture vs. antispasmodics in the treatment of irritable bowel syndrome: An adjusted indirect treatment comparison meta-analysis
Source: Front Physiol. 2022 Oct 6;13:1001978. doi: 10.3389/fphys.2022.1001978 (PMC9583016; doi:10.3389/fphys.2022.1001978)
Supplement: Supplementary file 1 [file Table1.DOCX]

Ovid MEDLINE(R) ALL <1946 to March 14, 2022>

1 randomised controlled trial.pt. 0

2 randomized controlled trial.pt. 561036

3 controlled clinical trial.pt. 94734

4 randomized.ab,ti. 600074

5 randomised.ab,ti. 119468

6 randomly.ab,ti. 378546

7 1 or 2 or 3 or 4 or 5 or 6 1237313

8 limit 7 to humans 982992

9 irritable bowel syndrome.ab,ti. 14272

10 ibs.ab,ti. 10046

11 ibs$.ab,ti. 11094

12 9 or 10 or 11 17392

13 parasympatholytics.ab,ti. 75

14 mebeverine.ab,ti. 177

15 alverine.ab,ti. 46

16 pinaverium.ab,ti. 141

17 rociverine.ab,ti. 34

18 cimetropium.ab,ti. 53

19 trimebutine.ab,ti. 236

20 drotaverine.ab,ti. 106

21 scopolamine derivative.ab,ti. 4

22 butylscopolamine.ab,ti. 151

23 hyoscine.ab,ti. 1069

24 muscarinic antagonists.ab,ti. 1301

25 pirenzepine.ab,ti. 3143

26 dicycloverine.ab,ti. 6

27 otilonium.ab,ti. 84

28 butylscopolammonium bromide.ab,ti. 23

29 acupuncture.ab,ti. 24529

30 acupuncture therapy.ab,ti. 1247

31 acupuncture points.ab,ti. 1328

32 acupuncture analgesia.ab,ti. 608

33 acu$.ab,ti. 1472065

34 electroacu$.ab,ti. 5255

35 13 or 14 or 15 or 16 or 17 or 18 or 19 or 20 or 21 or 22 or 23 or 24 or 25 or 26 or 27 or 28 or 29 or 30 or 31 or 32 or 33 or 34 1480058

36 limit 7 to clinical trial 290638

37 12 and 35 and 36 47

EMBASE Query Results Date

#36 #7 AND #12 AND #35 175 14-Mar-22

#35 #13 OR #14 OR #15 OR #16 OR #17 OR #18 OR #19 OR #20 OR #21 OR #22 OR #23 OR #24 OR #25 OR #26 OR #27 OR #28 OR #29 OR #30 OR #31 OR #32 OR #33 OR #34 43941 14-Mar-22

#34 electroacu$:ab,ti 6 14-Mar-22

#33 acu$:ab,ti 1835 14-Mar-22

#32 acupuncture analgesia':ab,ti 789 14-Mar-22

#31 acupuncture points':ab,ti 2128 14-Mar-22

#30 acupuncture therapy':ab,ti 1785 14-Mar-22

#29 acupuncture:ab,ti 34749 14-Mar-22

#28 butylscopolammonium bromide':ab,ti 28 14-Mar-22

#27 otilonium:ab,ti 118 14-Mar-22

#26 dicycloverine:ab,ti 13 14-Mar-22

#25 pirenzepine:ab,ti 3465 14-Mar-22

#24 muscarinic antagonists':ab,ti 1730 14-Mar-22

#23 hyoscine:ab,ti 1329 14-Mar-22

#22 butylscopolamine:ab,ti 206 14-Mar-22

#21 scopolamine derivative':ab,ti 3 14-Mar-22

#20 drotaverine:ab,ti 224 14-Mar-22

#19 trimebutine:ab,ti 365 14-Mar-22

#18 cimetropium:ab,ti 76 14-Mar-22

#17 rociverine:ab,ti 51 14-Mar-22

#16 pinaverium:ab,ti 245 14-Mar-22

#15 alverine:ab,ti 71 14-Mar-22

#14 mebeverine:ab,ti 272 14-Mar-22

#13 parasympatholytics:ab,ti 77 14-Mar-22

#12 #8 OR #9 OR #10 OR #11 36653 14-Mar-22

#11 ibs$:ab,ti 19499 14-Mar-22

#10 ibs:ab,ti 18236 14-Mar-22

#9 irritable bowel syndrome':ab,ti 22979 14-Mar-22

#8 irritable bowel syndrome'/exp 29462 14-Mar-22

#7 #6 AND ('human'/de OR 'randomized controlled trial'/de) AND 'Article'/it 712242 14-Mar-22

#6 #1 OR #2 OR #3 OR #4 OR #5 1116709 14-Mar-22

#5 controlled clinical trial':ab,ti 22085 14-Mar-22

#4 controlled clinical trial'/exp 878840 14-Mar-22

#3 randomized controlled trial':ab,ti 118223 14-Mar-22

#2 randomized controlled trials as topic'/exp 222133 14-Mar-22

#1 randomized controlled trial'/exp 702816 14-Mar-22

CENTRAL (Cochrane Central Register of Controlled Trials)

Search Name:

Date Run: 14/03/2022 10:32:06

Comment:

ID Search Hits

#1 MeSH descriptor: [Randomized Controlled Trial] explode all trees 119

#2 (randomized controlled trial):ti,ab,kw (Word variations have been searched) 723334

#3 MeSH descriptor: [Controlled Clinical Trial] explode all trees 128

#4 (controlled clinical trial):ti,ab,kw (Word variations have been searched) 613356

#5 #1 OR #2 OR #3 OR #4 in Trials 771984

#6 MeSH descriptor: [Irritable Bowel Syndrome] explode all trees 1331

#7 (irritable bowel syndrome):ti,ab,kw (Word variations have been searched) 4117

#8 (IBS):ti,ab,kw (Word variations have been searched) 6371

#9 (IBS$):ti,ab,kw (Word variations have been searched) 6371

#10 #6 OR #7 OR #8 OR #9 7713

#11 (eluxadoline):ti,ab,kw (Word variations have been searched) 60

#12 (parasympatholytics):ti,ab,kw (Word variations have been searched) 831

#13 (mebeverine):ti,ab,kw (Word variations have been searched) 94

#14 (alverine):ti,ab,kw (Word variations have been searched) 28

#15 (pinaverium):ti,ab,kw (Word variations have been searched) 112

#16 (rociverine):ti,ab,kw (Word variations have been searched) 11

#17 (cimetropium):ti,ab,kw (Word variations have been searched) 42

#18 (trimebutine):ti,ab,kw (Word variations have been searched) 112

#19 (drotaverine):ti,ab,kw (Word variations have been searched) 59

#20 (scopolamine derivatives):ti,ab,kw (Word variations have been searched) 412

#21 (butylscopolamine):ti,ab,kw (Word variations have been searched) 47

#22 (hyoscine):ti,ab,kw (Word variations have been searched) 497

#23 (trimebutine):ti,ab,kw (Word variations have been searched) 112

#24 (muscarinic antagonists):ti,ab,kw (Word variations have been searched) 1860

#25 (pirenzepine):ti,ab,kw (Word variations have been searched) 676

#26 (dicyclomine):ti,ab,kw (Word variations have been searched) 57

#27 (otilonium):ti,ab,kw (Word variations have been searched) 37

#28 (butylscopolammonium bromide):ti,ab,kw (Word variations have been searched) 214

#29 (acupuncture):ti,ab,kw (Word variations have been searched) 16198

#30 (acupuncture therapy):ti,ab,kw (Word variations have been searched) 9018

#31 (acupuncture points):ti,ab,kw (Word variations have been searched) 5945

#32 (acupuncture analgesia):ti,ab,kw (Word variations have been searched) 1142

#33 (acu$):ti,ab,kw (Word variations have been searched) 306

#34 (electroacu$):ti,ab,kw (Word variations have been searched) 2

#35 #11 OR #12 OR #13 OR #14 OR #15 OR #16 OR #17 OR #18 OR #19 OR #20 OR #21 OR #22 OR #23 OR #24 OR #25 OR #26 OR #27 OR #28 OR #29 OR #30 OR #31 OR #32 OR #33 OR #34 20799

#36 #5 AND #10 AND #35 253
