# Supplementary material for: Acupuncture vs. antispasmodics in the treatment of irritable bowel syndrome: An adjusted indirect treatment comparison meta-analysis
Source: Front Physiol. 2022 Oct 6;13:1001978. doi: 10.3389/fphys.2022.1001978 (PMC9583016; doi:10.3389/fphys.2022.1001978)
Supplement: Supplementary file 2 [file Table2.DOCX]

**Author(s):** Yun-zhou Shi, Qing-feng Tao
**Date:** 2022-07-24
**Question:** Should acupuncture vs antispasmodics be used for IBS?
**Settings:**
**Bibliography:**

| **Quality assessment** | | | | | | | **No of patients** | | **Effect** | | **Quality** | **Importance** |
| --- | --- | --- | --- | --- | --- | --- | --- | --- | --- | --- | --- | --- |
|  |  |  |  |  |  |  |  |  |  |  |  |  |
| **No of studies** | **Design** | **Risk of bias** | **Inconsistency** | **Indirectness** | **Imprecision** | **Other considerations** | **Acupuncture** | **Antispasmodics** | **Relative (95% CI)** | **Absolute** |  |  |
| **abdominal pain** | | | | | | | | | | | | |
| 9 | randomised trials | serious^1^ | serious^2^ | Serious^3^ | no serious imprecision | none | - | - | - | - | ⊕OOO VERY LOW | CRITICAL |
|  |  |  |  |  |  |  |  | 0% |  | - |  |  |
| **the relief of Global IBS symptoms** | | | | | | | | | | | | |
| 16 | randomised trials | serious^1^ | serious^2^ | Serious^3^ | no serious imprecision | none | - | - | - | - | ⊕OOO VERY LOW | IMPORTANT |
|  |  |  |  |  |  |  |  | 0% |  | - |  |  |
| **adverse events** | | | | | | | | | | | | |
| 17 | randomised trials | serious^1^ | no serious inconsistency | Serious^3^ | no serious imprecision | none | - | - | - | - | ⊕⊕OO LOW | IMPORTANT |
|  |  |  |  |  |  |  |  | 0% |  | - |  |  |

^1^Method of randomization is unclear or lack of .
^2^The variety of antispasmodics including different types, dosages, usages, and duration of treatment and different acupuncture prescriptions maybe contribute to the statistical heterogeneity.

^3^ Indirect comparison.
